# Exposure to the natural alkaloid Berberine affects cardiovascular system morphogenesis and functionality during zebrafish development

**DOI:** 10.1038/s41598-020-73661-5

**Published:** 2020-10-15

**Authors:** Davide Martini, Cecilia Pucci, Chiara Gabellini, Mario Pellegrino, Massimiliano Andreazzoli

**Affiliations:** 1 Cell and Developmental Biology Unit, Department of Biology, University of Pisa, SS12 Abetone e Brennero, 56127 Pisa, Italy; 2 Sant’Anna School of Advanced Studies, Pisa, Italy; 3 National Institute of Optics, National Research Council, Pisa, Italy; 4 Interdepartmental Research Center Nutrafood “Nutraceuticals and Food for Health”, University of Pisa, Pisa, Italy; 5 Present Address: Institute of Genomic Medicine, Catholic University, 00168 Rome, Italy

**Keywords:** Angiogenesis, Zebrafish, Heart development

## Abstract

The plant-derived natural alkaloid berberine displays therapeutic potential to treat several pathological conditions, including dyslipidemias, diabetes and cardiovascular disorders. However, data on berberine effects during embryonic development are scarce and in part controversial. In this study, using zebrafish embryos as vertebrate experimental model, we address the effects of berberine treatment on cardiovascular system development and functionality. Starting from the observation that berberine induces developmental toxicity and pericardial edema in a time- and concentration-dependent manner, we found that treated embryos display cardiac looping defects and, at later stages, present an abnormal heart characterized by a stretched morphology and atrial endocardial/myocardial detachment. Furthermore, berberine affected cardiac functionality of the embryos, promoting bradycardia and reducing the cardiac output, the atrial shortening fraction percentage and the atrial stroke volume. We also found that, during development, berberine interferes with the angiogenic process, without altering vascular permeability. These alterations are associated with increased levels of vascular endothelial growth factor aa (*vegfaa)* mRNA, suggesting an important role for Vegfaa as mediator of berberine-induced cardiovascular defects. Altogether, these data indicate that berberine treatment during vertebrate development leads to an impairment of cardiovascular system morphogenesis and functionality, suggesting a note of caution in its use during pregnancy and lactation.

## Introduction

Plant-derived natural compounds are important sources of medicinal agents to design new therapeutic strategies and are commonly considered effective and safe^1^. However, a large body of evidence indicates that natural compounds may affect several biological processes and promote various deleterious effects, including genotoxicity, carcinogenicity and teratogenicity^2,3^, suggesting the need for in-depth studies and validated trials of natural substances. Berberine (BRB), 5,6-dihydro-9,10-dimethoxy-benzo(g)-1,3-benzodioxolo(5,6-α)quinolizinium, is a natural isoquinoline alkaloid and one of the major active components isolated from various plants belonging to Berberidaceae, Papaveraceae and Ranunculaceae family. Extracts from these plants, and BRB itself, are traditionally used in the Chinese and Ayurvedic medicine to treat various kinds of illnesses. More recently, several bioactive and pharmacological properties of BRB have been extensively demonstrated, indicating its enormous therapeutic potential^4–6^. In clinical applications, BRB is usually administered orally as sulphate or chloride formulation at 400–1500 mg/day^6,7^ and exerts multi-target protective action against several pathological conditions, including dyslipidemias, diabetes, arrhythmias, heart failure, cancer and neurodegenerative disorders^4–6^. In particular, BRB is already available in over-the-counter dietary supplements for the treatment of type 2 diabetes mellitus and dyslipidemia^8,9^.

BRB regulates multiple signaling pathways, and influences several cellular processes, including migration, proliferation, apoptosis and gene expression^4,6,10–14^. Studies in rats and humans showed that BRB can be absorbed from the gastrointestinal tract after oral administration, although its bioavailability and plasma levels are very low. However, results in rats suggested that the high effectiveness of BRB may stem from the fact that it is distributed and accumulates in various organs, including the heart, reaching concentrations higher than those in the plasma^15^. Although BRB is considered a compound with a good safety profile and a high therapeutic potential, a certain degree of toxicity, genotoxicity, mutagenicity, carcinogenicity and cardiotoxicity of BRB have been reported in several experimental models in vitro and in vivo, indicating that the safety of this nutraceutical is strictly dependent on the experimental model, the route and duration of administration, as well as the concentration/dose used^6^. However, BRB toxicity has been also reported in clinical experience. For instance, gastrointestinal problems have been documented in patients with type-2 diabetes orally administered with BRB^16^. In addition, BRB infusion promoted severe cardiovascular functionality alterations in patients with congestive heart failure^17^. Notably, developmental toxicity of BRB is a poorly studied aspect that still remains a controversial topic of discussion, although a limited number of works have already suggested that BRB treatment should be avoided during pregnancy and lactation^6^. For instance, oral administration of BRB in rodent mothers during gestation reduced the average fetal body weight per litter and induced fetal malformations^18^. In addition, BRB inhibited the development of mouse zygotes to blastocysts in vitro and reduced the percentage of recovered blastocysts and fully developed fetuses when intramuscularly administered in mated superovulated mice in vivo^19,20^. Furthermore, previous results in vitro and in vivo showed that BRB promotes oxidative stress-mediated apoptosis in mouse developing embryos and indicated that BRB impairs embryonic implantation and the development potential of post-implantation embryos^21^. Moreover, Ali and colleagues showed that addition of BRB to the culturing medium of zebrafish embryos from 24 h post-fertilization (hpf) to 120 hpf resulted in increased mortality rate in a time- and concentration-dependent manner, and in phenotypic abnormalities, such as uninflated swim bladder, yolk sac edema and pericardial edema, in a concentration-dependent manner^22–24^. On the other hand, other works indicated that BRB did not induce developmental toxicity, as previously shown in vitro in mouse^25^ and porcine early embryos^26^. In this line, BRB increased clinical pregnancy rate and promoted the survival away from the nest following the transplantation of in vitro developed early mouse embryos^25^ and continuous intragastric administration of Chinese Goldthread rhizome decoction, which contains BRB, or pure BRB to pregnant rats and mice did not cause abortion and developmental toxicity^27^.

BRB appears to have also an effect on the cardiovascular system, which is one of the first organ system to form and function during vertebrate embryonic development and that maintains morphological and genetic plasticity also in the adult^28–30^. Vascular endothelial growth factor-A (VEGF-A) and vascular endothelial cadherin (Cdh5) are key factors that critically regulate cardiovascular system development and functionality in vertebrates^28–32^. Interestingly, several results indicated that BRB may influence VEGF-A at both mRNA and protein level as shown, for instance, in various cultured human cancer cell lines^12–14^ and in murine tumors^14^. In contrast, data on BBR effects on Cdh5 are very limited at present. BRB treatment has been reported to counteract the reduction of Cdh5 protein levels induced in vitro by lipopolysaccharide in rat intestinal microvascular endothelial cells^33^. In addition, the BRB-type natural alkaloid Coptisine inhibited the expression of Cdh5 in vitro in a human osteosarcoma cell line, at both mRNA and protein level^34^. Moreover, previous results suggested that BRB may modulate the angiogenic process during development, as reported, for instance, in murine embryonic stem cell-derived embryoid bodies^35,36^ and developing zebrafish embryos^14^.

Zebrafish is largely considered as a high-throughput and alternative vertebrate experimental model in preclinical drug screening, safety pharmacology, cardiotoxicity and developmental toxicity assessment, and is characterized by a good predictivity for the toxicity and teratogenicity of various substances in mammals^6,22–24,37^. In addition, zebrafish is a well-known and widely accepted experimental model to study the cardiovascular system development and functionality in vertebrates, both in physiological and pathological conditions^28,29,38^.

In this work, we describe how exposure to BRB induces developmental toxicity and, in particular, it affects embryonic cardiovascular system development and functionality in zebrafish.

## Results

### Berberine exerts toxic and teratogenic effects in developing zebrafish embryos

**Figure 1:**
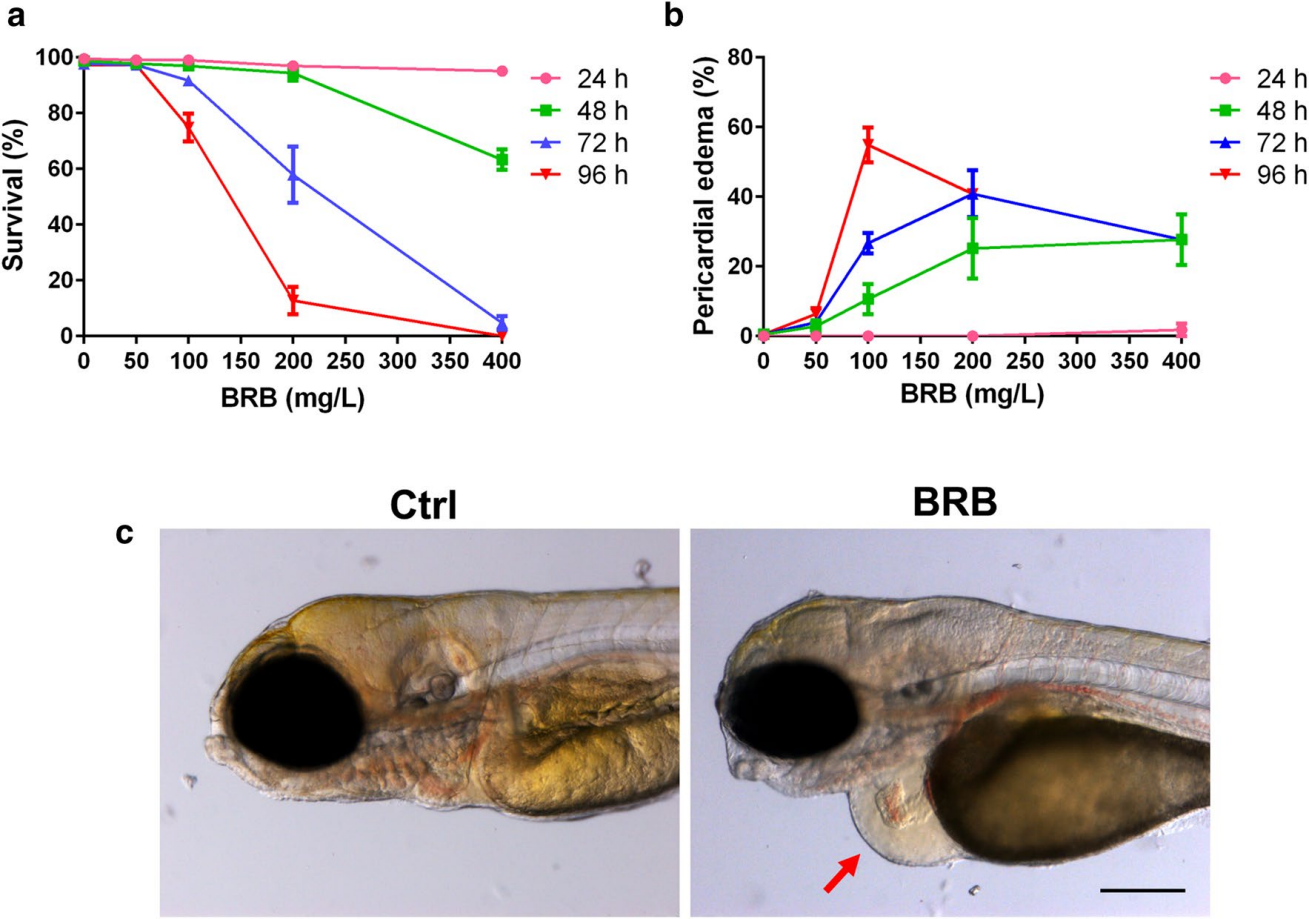
Toxicological and teratogenic effects of BRB on zebrafish development. (**a**) Survival rate and (**b**) percentage of larvae with pericardial edema, evaluated in zebrafish exposed from 24 hpf either to control conditions (Hank’s buffer) or to increasing concentrations of BRB (50, 100, 200 and 400 mg/L) for 24, 48, 72 and 96 h. Data are cumulative and expressed as mean ± standard error of the mean (SEM) from 5 independent experiments, each with 40 larvae per experimental condition. (**c**) Representative images of 72 hpf zebrafish larvae exposed to control conditions (Ctrl) showing the normal embryonic phenotype (left) and the altered phenotype characterized by the formation of the pericardial edema (red arrow) reported in BRB treated animals (right); lateral views, anterior to the left. Scale bar: 200 µm. Images were created using GraphPad PRISM version 6.0 (https://www.graphpad.com/support/), Photoshop version CS5 (https://www.adobe.com/products/photoshop.html) and PowerPoint version 16.16.24 (https://support.microsoft.com/en-us/powerpoint).

In the present study, the zebrafish pigmentless casper (*roy*^*a9/a9*^*;nacre*^*w2/*w2^) line was used because its improved transparency facilitates the analysis of the cardiovascular system development and physiology, also at later developmental stages. We initially evaluated the effects of increasing concentrations of BRB (50, 100, 200 and 400 mg/L) on survival rate (Fig. 1a, Supplementary Table S1) and induction of pericardial edema (Fig. 1b,c, Supplementary Table S2) in zebrafish embryos at different developmental stages (48, 72, 96, and 120 hpf), starting the treatment at 24 hpf. Compared to the embryos exposed to control conditions, embryos survival and their gross morphology were not significantly influenced by the incubation with 50 mg/L BRB, independently from the time-point exposure, as well as by 24 h of BRB exposure at any concentration. In contrast, BRB exposure for 48 h significantly reduced the embryo survival at 400 mg/L (~ 63.3%) and, starting from 100 mg/L, promoted a significant increase in the incidence of embryos with pericardial edema (~ 12.1%, 25.2% and 27.7% at 100, 200 and 400 mg/L, respectively). After 72 h of exposure, BRB treatment significantly reduced the survival rate at 200 mg/L (~ 57.9%), resulted lethal for almost all embryos at 400 mg/L and further increased the incidence of embryos with pericardial edema at 100 (~ 26.7%) and 200 mg/L (~ 40.8%). Finally, at 96 h of exposure, BRB treatment effects were further intensified. In particular, the survival rate was significantly reduced also at 100 mg/L (~ 74.8%), further decreased at 200 mg/L (~ 12.8%) whereas the incidence of embryos with pericardial edema still increased at 100 mg/L (~ 54.9%). In line with previous works^22,23^, we calculated the concentrations that caused the death of 50% of the embryos (LC_50_) after 72 h and 96 h of exposure, which were found to correspond to 213 ± 8.03 mg/L and 156 ± 4.96 mg/L, respectively. Therefore, for subsequent investigations, we mostly used the concentration of 100 mg/L BRB as it was lower than the LC_50_ value after 72 h and 96 h of exposure and resulted to be the lowest concentration able to promote pericardial edema with minimal effects on embryo survival.

**Figure 2:**
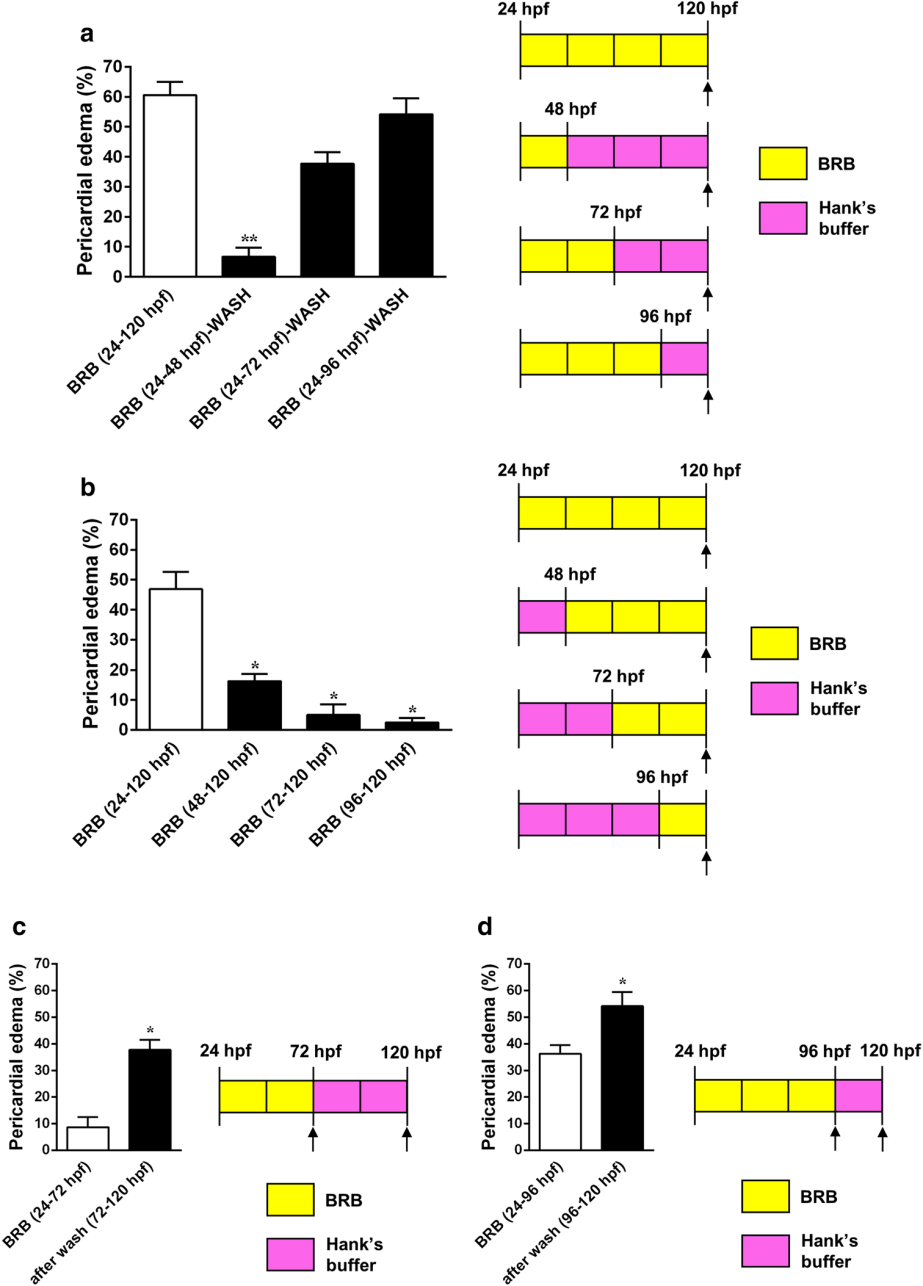
Critical time window for the teratogenic action of BRB (**a**,**b**) and effects of wash-out (**c**,**d**). (**a**) Percentage of larvae with pericardial edema at 120 hpf, previously exposed to 100 mg/L BRB from 24 hpf for different exposure durations (24, 48, 72 h), successively washed and incubated in Hank’s buffer for the remaining time. Zebrafish larvae exposed to 100 mg/L from 24 to 120 hpf were considered as internal control (white column). (**b**) Percentage of larvae with pericardial edema at 120 hpf, previously exposed to 100 mg/L BRB from different developmental stages (24 hpf, 48 hpf, 72 hpf, 96 hpf) to 120 hpf. Zebrafish larvae exposed to 100 mg/L BRB from 24 to 120 hpf were considered as internal control (white column). (**a**,**b**) Data are cumulative and expressed as mean ± SEM from 4 independent experiments, each with 40 larvae per experimental condition. Statistical analysis was performed by Kruskal–Wallis test followed by Dunn’s multiple comparison test. *p < 0.05 and **p < 0.01 vs control (BRB 24–120 hpf). (**c**,**d**) Percentage of larvae with pericardial edema, previously exposed to 100 mg/L BRB from 24 hpf for (**c**) 48 h and (**d**) 72 h, successively washed and incubated in Hank’s buffer until 120 hpf. Cumulative data were calculated at the end of the period of BRB exposure (white columns) as well as at 120 hpf (black columns) and are expressed as mean ± SEM from 4 independent experiments, each with 40 larvae for each experimental condition. Statistical analysis was performed by Mann–Whitney U test. *p < 0.05 vs the value at the end of the period of incubation in BRB. Images were created using GraphPad PRISM version 6.0 (https://www.graphpad.com/support/) and PowerPoint version 16.16.24 (https://support.microsoft.com/en-us/powerpoint).

In order to identify the critical window of exposure to sub-lethal concentrations of BRB that promoted the maximum incidence of embryos with pericardial edema, we performed a series of experiments in which 100 mg/L BRB was applied at 24 hpf, for different exposure durations, followed by washes, incubation in control medium and quantification at 120 hpf. As shown in Fig. 2a, the incidence of embryos developing pericardial edema appeared to be dependent on the exposure duration when compared to the value obtained when BRB was continuously applied from 24 to 120 hpf. In particular, compared to the continuous treatment with 100 mg/L BRB from 24 to 120 hpf, the treatment with BRB from 24 to 48 hpf resulted in a significative reduction (~ 89%) in the percentage of embryos with pericardial edema at 120 hpf, whereas the other treatments did not generate significative differences, although the general trend shows an effect dependent on the exposure duration. These results suggest that the last 48 h of BRB exposure (from 72 and 96 hpf to 120 hpf) have a more limited impact on the teratogenic effects of BRB.

Additionally, we evaluated the effects on the incidence of pericardial edema when embryos were exposed to 100 mg/L BRB starting the treatment at different developmental stages and incubating the embryos until 120 hpf (quantification at 120 hpf). As shown in Fig. 2b, no significant difference was observed between BRB treatment starting from 48 hpf, 72 hpf and 96 hpf, although the trend is suggestive again of an effect dependent on treatment duration. However, the percentage of embryos with pericardial edema obtained in these experimental conditions were significantly reduced compared to the value reported when BRB was continuously applied from 2 to 120 hpf, suggesting that during the first 24 h of development the embryos are particularly sensitive to the teratogenic effect of BRB.

Furthermore, we treated embryos with 100 mg/L BRB applied from 24 hpf to either 72 hpf or 96 hpf, followed by washes and incubation in control medium for the remaining time until 120 hpf. In these experiments, we evaluated, and compared to each other, the percentage of embryos developing pericardial edema in two distinct moments: immediately at the end of BRB treatment and at 120 hpf. Compared to the values obtained after BRB exposure from 24 to 72 hpf (Fig. 2c) or 96 hpf (Fig. 2d), the incidence of embryos with pericardial edema at 120 hpf resulted increased by ~ 3.4-fold and ~ by 49.3%, respectively, suggesting that the teratogenic action triggered by BRB exposure continued to occur even after the alkaloid was removed from the environment.

### BRB alters heart looping, but not the markers of cardiac valves morphogenesis, during early stages of zebrafish embryonic development

**Figure 3:**
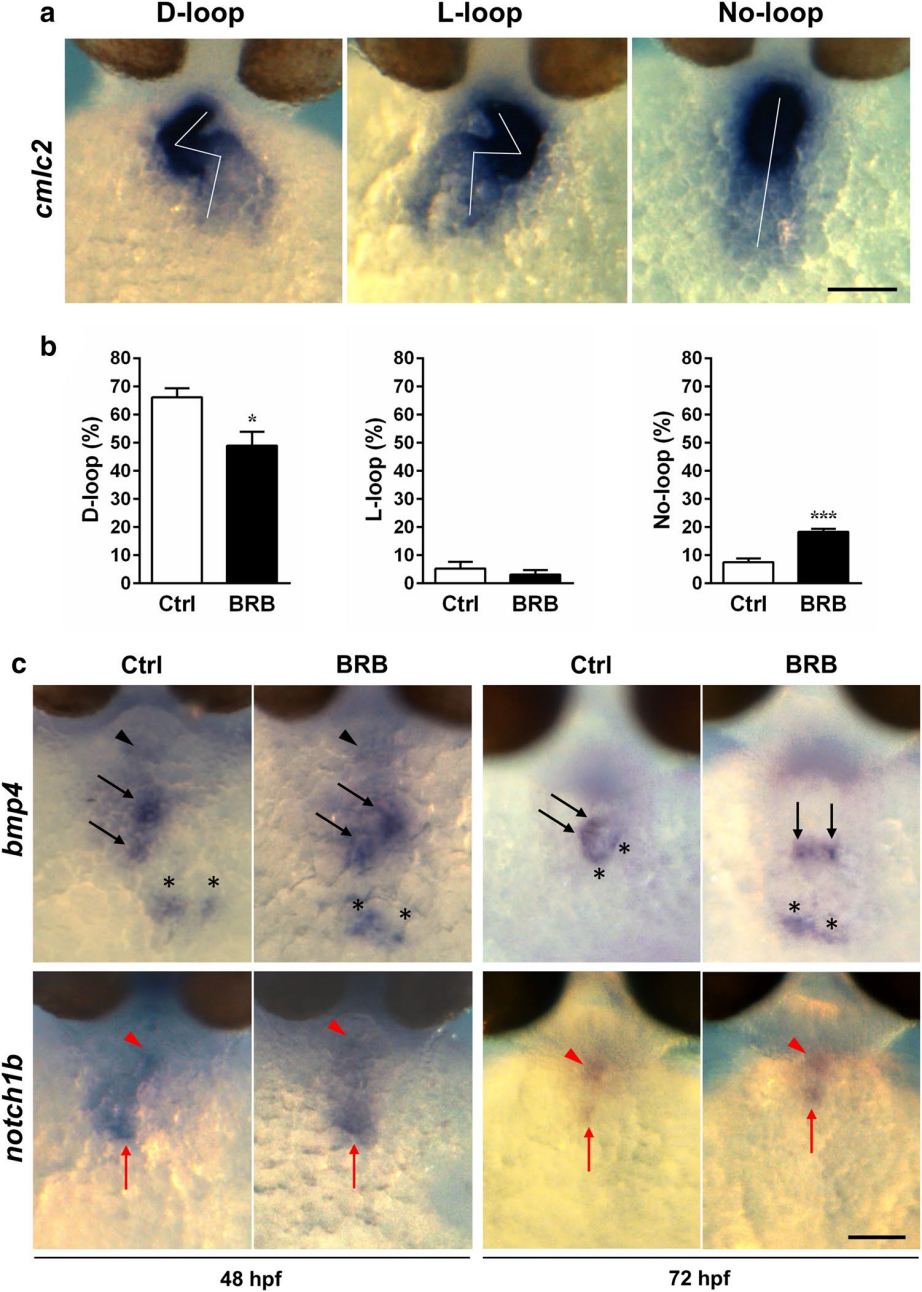
Effects of BRB on early heart morphogenesis in zebrafish larvae. (**a**) Representative images of heart morphology (normal phenotype “D-loop” and abnormal phenotypes “L-loop” and “No-loop”) obtained by WISH for *cmlc2* (*myl7*) in zebrafish larvae at 48 hpf previously treated for 24 h either in control conditions (Hank’s buffer) or in the presence of 100 mg/L BRB; ventral views, anterior to the top. (**b**) Percentage of embryos with different heart-looping direction (D-loop, L-loop and No-loop) in zebrafish larvae at 48 hpf, previously incubated for 24 h either in control conditions (Ctrl) or in the presence of 100 mg/L BRB. Data are expressed as mean ± SEM from 5 independent experiments, each with 25 larvae per experimental condition. Statistical analysis was performed by Student’s t-test. *p < 0.05 and ***p < 0.001 vs control. (**c**) Representative images obtained by WISH for *bmp4* and *notch1b* in zebrafish larvae at 48 hpf and 72 hpf, previously treated for 24 h and 48 h, respectively, either in control conditions (Ctrl) or in the presence of 100 mg/L BRB; ventral views, anterior to the top. Images show that *bmp4* is expressed in the myocardium at the level of cardiac outflow tract (black arrowhead), atrioventricular ring (black arrows) and cardiac inflow tract (black asterisks) whereas *notch1b* is expressed in the endocardium of cardiac outflow tract (red arrowhead) and atrioventricular ring (red arrow). (**a**,**c**) Scale bar: 75 µm. Images were created using GraphPad PRISM version 6.0 (https://www.graphpad.com/support/), Photoshop version CS5 (https://www.adobe.com/products/photoshop.html) and PowerPoint version 16.16.24 (https://support.microsoft.com/en-us/powerpoint).

Pericardial edema in zebrafish embryos can be caused by early morphological defects such as uncorrected cardiac looping and/or valve development^32,39,40^. To study the effects on cardiac looping, we performed whole mount in situ hybridization (WISH) in 48 hpf zebrafish embryos previously incubated in control conditions or in 100 mg/L BRB for 24 h, using antisense probes directed against cardiac myosin light chain 2 (*cmlc2)*, a robust marker of differentiated cardiomyocytes. As shown in Fig. 3a, *cmlc2* expression was localized in both heart chambers of 48 hpf embryos treated in control conditions or exposed to BRB. Our quantitative analysis of the incidence of the main cardiac morphologies found (Fig. 3b) showed that the heart of most embryos exposed to control conditions developed the correct S-shaped loop (D-loop), with the ventricle positioned along the midline and the atrium on the left below, whereas the incidence of embryos with abnormal cardiac looping phenotypes, such as L-loop and no-loop, was minimal. As shown in Fig. 3b, the treatment with 100 mg/L BRB determined a significant reduction in the incidence of embryos with normal D-loop cardiac phenotype (~ 26% vs control) and a significant increase of the percentage of embryos with no-loop heart phenotype (~ 144.1% vs control) whereas there were no significant effects on the incidence of embryos with L-loop cardiac morphology.

To investigate the effects of BRB on cardiac valves development, we analyzed by WISH the expression pattern of two well-known markers critically involved in the early valve morphogenesis, such as *notch1b* and *bmp4*, which are expressed at endocardial level and myocardial level, respectively. As shown in Fig. 3c, we did not observe significant differences in the expression of both *bmp4* and *notch1b* between BRB-treated larvae and control larvae at both 48 and 72 hpf, with the exception of the obvious variations due to the different heart morphology of embryos with defective cardiac looping, particularly visible at 72 hpf*.*

### BRB impairs late heart morphogenesis in zebrafish larvae

**Figure 4:**
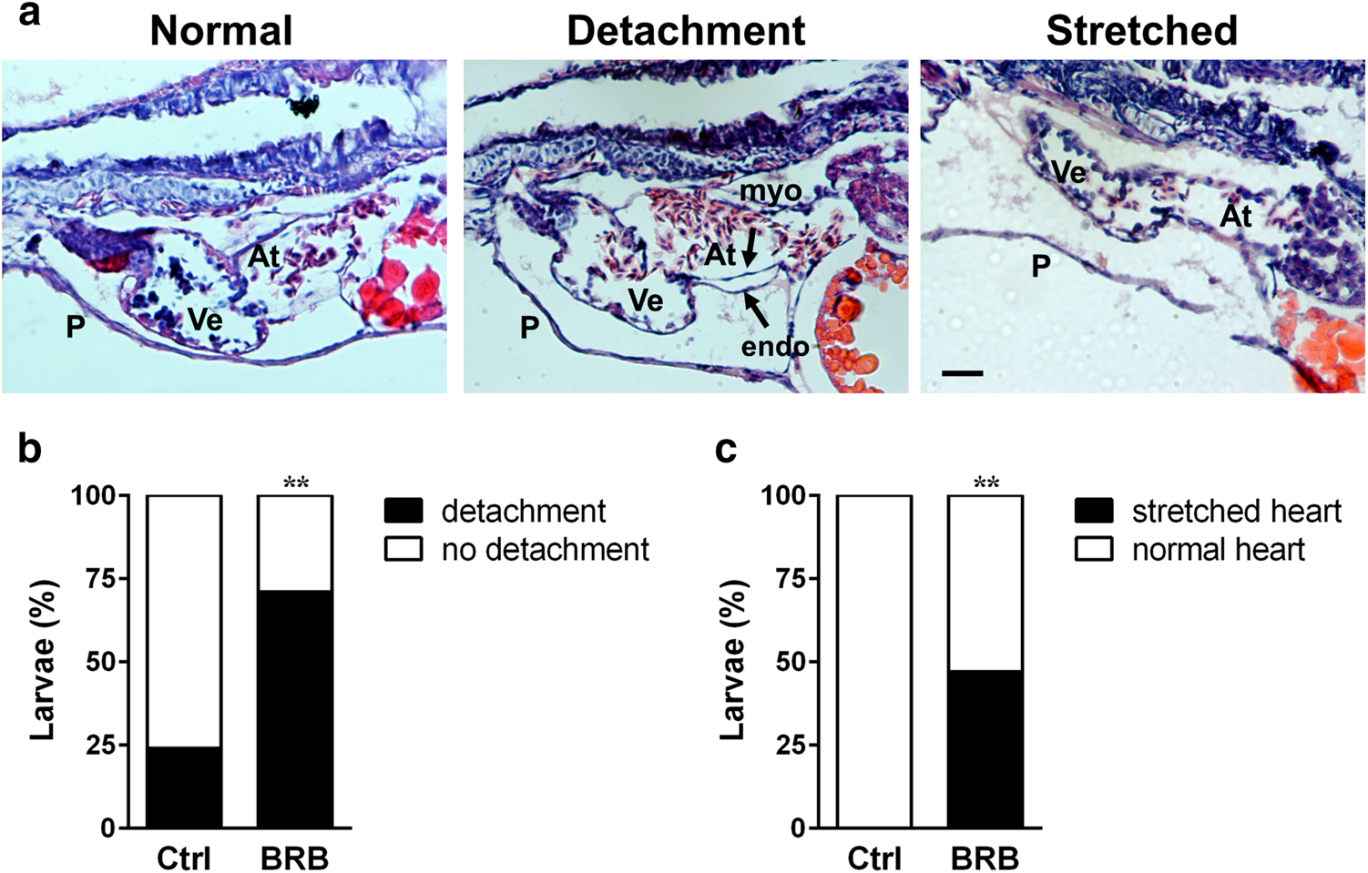
Effects of BRB on late heart morphogenesis in zebrafish larvae. (**a**) Representative images of H&E-stained sagittal histological sections of zebrafish larvae at 120 hpf, previously treated for 96 h either in control conditions (Hank’s buffer; Ctrl) or in the presence of 100 mg/L BRB. The images show the myocardium-endocardium detachment in the atrium (black arrows) and the stretched heart, compared with the normal heart phenotype. *At* atrium, *Ve* ventricle, *P* pericardium, *myo* myocardium, *endo* endocardium. Lateral views, anterior to the left; scale bar: 25 µm. (**b**,**c**) Quantification of the percentage of embryos with atrial myocardium-endocardium detachment (**b**) and with stretched heart (**c**). Data are calculated from 18 to 19 larvae for each experimental condition, obtained from 3 independent experiments. Statistical analysis was performed on raw data by Fisher test, and then represented as percentage. **p < 0.01 vs control. Images were created using GraphPad PRISM version 6.0 (https://www.graphpad.com/support/), Photoshop version CS5 (https://www.adobe.com/products/photoshop.html) and PowerPoint version 16.16.24 (https://support.microsoft.com/en-us/powerpoint).

To determine whether BRB-induced pericardial edema in zebrafish larvae was associated with structural defects in late heart morphogenesis, we examined hematoxylin–eosin-stained sections of larvae at 120 hpf, previously exposed to either control conditions or 100 mg/L BRB for 96 h. Compared to control embryos, BRB treated larvae displayed an increased incidence (~ 196% vs control) of an abnormal atrial endocardial/myocardial detachment (Fig. 4a,b) and of a stretched heart morphology (~ 47%) (Fig. 4a,c). The morphology of other cardiac structures, including AV valves, bulbus arteriosus and venous sinus did not seem to be affected by BRB treatment.

### BRB affects early cardiac functionality during zebrafish development

**Figure 5:**
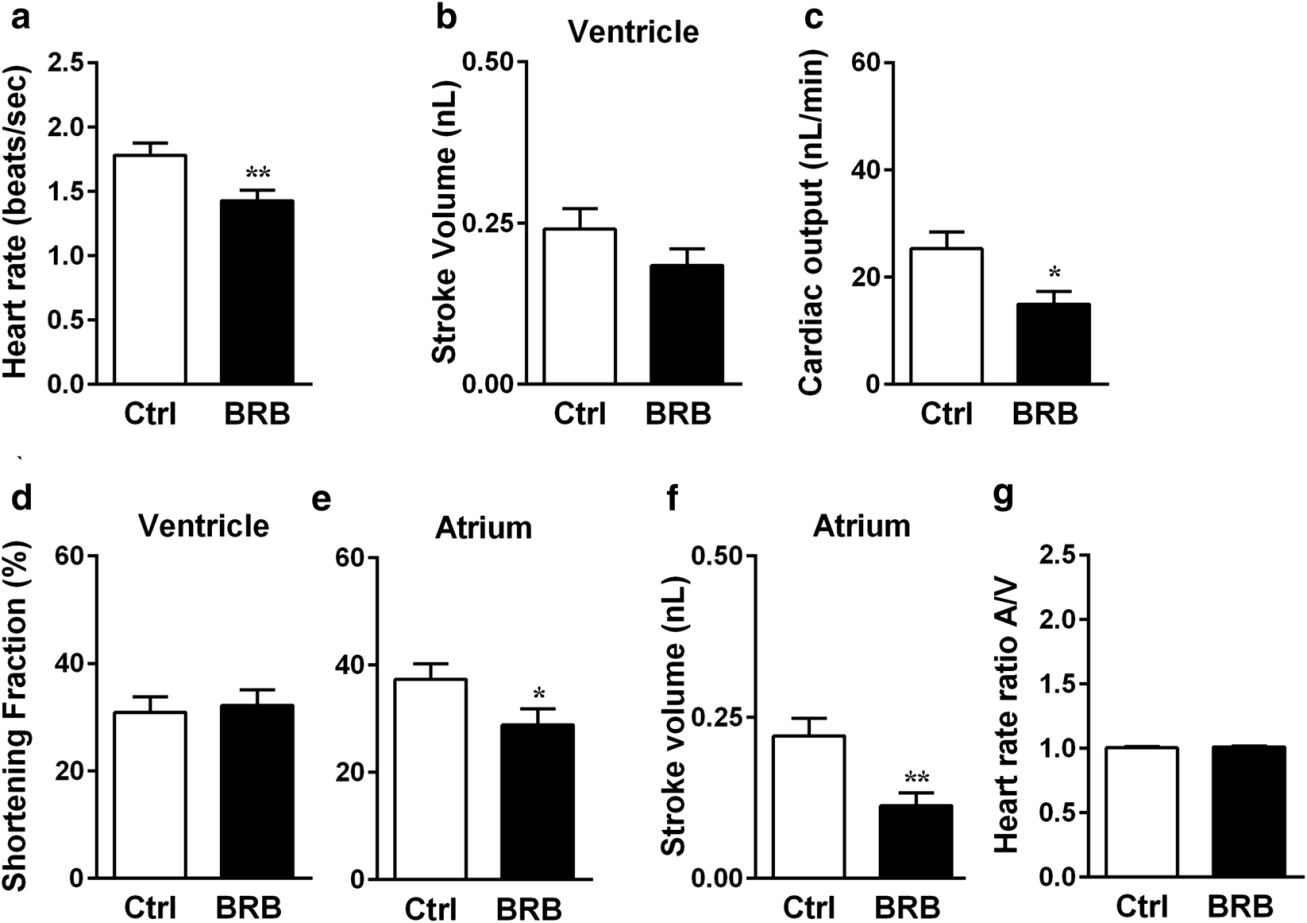
Effects of BRB on early cardiac functionality of developing zebrafish embryos. Quantification of heart rate (**a**), ventricular stroke volume (**b**), cardiac output (**c**), ventricular (**d**) and atrial (**e**) shortening fraction percentage, atrial stroke volume (**f**) and A/V rates ratio (**g**) in 48 hpf zebrafish embryos of the Tg(*kdrl*:EGFP)^S843^/*roy*^*a9/a9*^*;nacre*^*w2/w2*^ casper line previously treated for 24 h either in control conditions (Hank’s buffer; Ctrl) or in the presence of 100 mg/L BRB. Data are expressed as mean ± SEM from 24 larvae for each experimental condition, obtained from 3 independent experiments. Statistical analysis was performed by Student’s t-test. *p < 0.05 and **p < 0.01 vs control. Images were created using GraphPad PRISM version 6.0 (https://www.graphpad.com/support/).

Impairment of heart functionality during early developmental stages of zebrafish and other fishes may be responsible for pericardial edema formation at later stages^32,41^. To study the effects of BRB on early cardiac functionality we evaluated various parameters, such as heart rate, atrial and ventricular systolic stroke volume, cardiac output, ventricular and atrial shortening fraction percentage and A/V rates ratio. To visualize beating heart in living embryos, we analyzed 48 hpf zebrafish embryos of the transgenic casper line Tg(*kdrl*:EGFP)^S843^/*roy*^*a9/a9*^*;nacre*^*w2/w2*^, which expresses EGFP in vascular endothelial cells and endocardium, comparing control embryos with embryos previously exposed to 100 mg/L BRB for 24 h. As shown in Fig. 5a, the heart beating rate in control embryos was ~ 1.78 beats/s, and BRB exposure significantly decreased heart rate to ~ 1.42 beats/s. Nevertheless, there was no significant difference between control and BRB treated larvae in ventricular stroke volume (Fig. 5b) and ventricular shortening fraction percentage (Fig. 5d). Cardiac output was reduced by ~ 37% in BRB-treated larvae compared to control (Fig. 5c), suggesting that the impairment of cardiac output elicited by BRB treatment was due to the reduced heart rate. On the other hand, BRB treatment determined a significant reduction of atrial shortening fraction percentage (Fig. 5e) and atrial stroke volume (Fig. 5f) of ~ 20% and ~ 41%, respectively, compared to control conditions. Finally, as shown in Fig. 5g, A/V rates ratio was 1:1, both in control and BRB treated larvae, indicating that BRB-induced bradycardia was not associated with atrio-ventricular conduction impairments.

### BRB treatment causes vascular defects during zebrafish development

**Figure 6:**
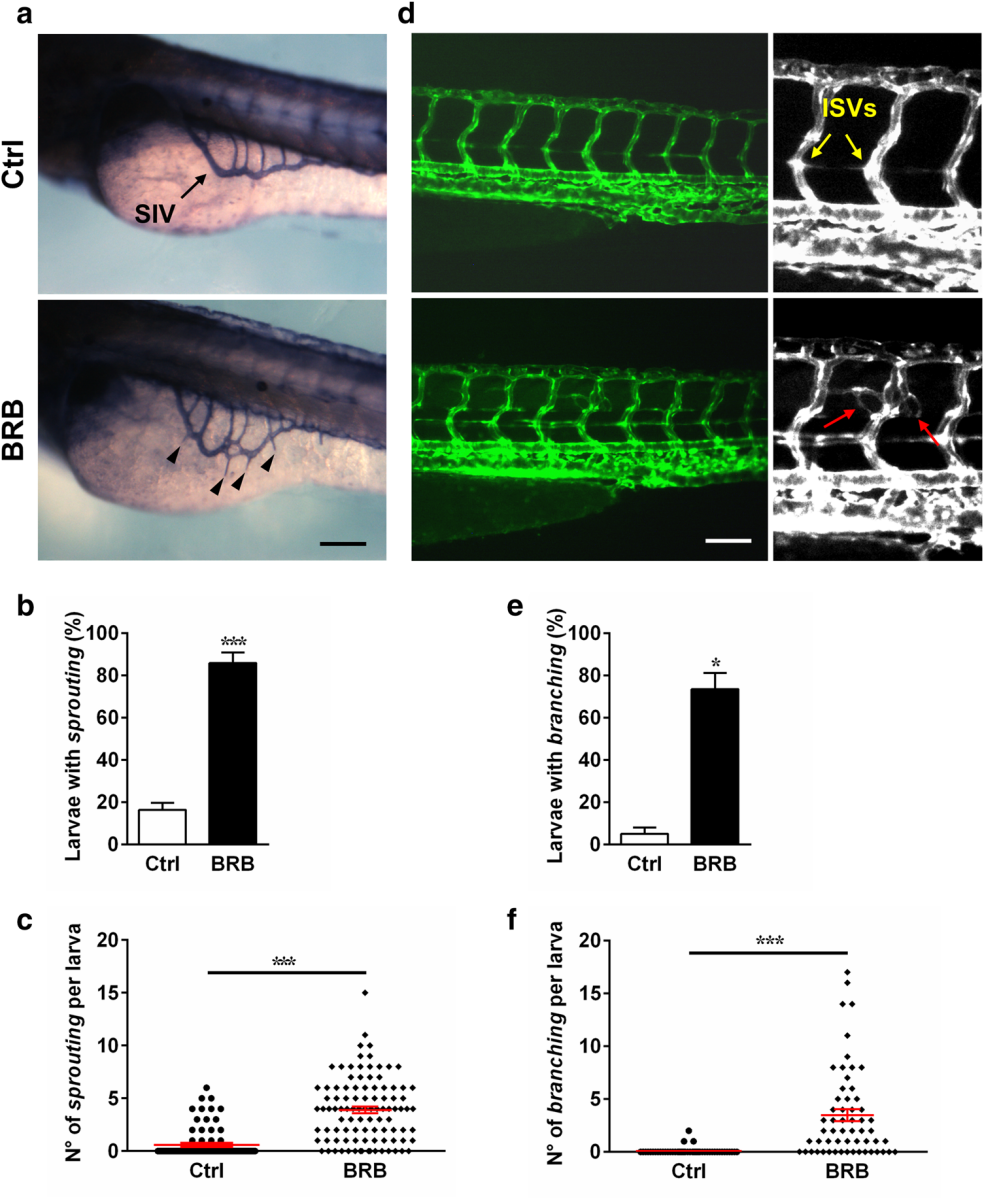
Effects of BRB on developing SIV (**a**–**c**) and ISVs (**d**–**f**) in zebrafish larvae. (**a**) Representative images of SIV evidenced by whole-mount alkaline phosphatase staining in zebrafish larvae at 72 hpf previously treated for 48 h either in control conditions (Hank’s buffer; Ctrl) or in the presence of 100 mg/L BRB; black arrowheads indicate the sprouting vessels. Quantification of the percentage of zebrafish larvae with sprouting vessels (**b**) and of the number of sprouting per larva (**c**). (**d**) Representative images of ISVs observed in 72 hpf Tg(*kdrl*:EGFP)^S843^/*roy*^*a9/a9*^*;nacre*^*w2/w2*^casper zebrafish larvae previously treated for 48 h either in control conditions (top row) or in the presence of 100 mg/L BRB (bottom row); red arrows indicate the branching vessels. Quantification of the percentage of zebrafish larvae with branching (**e**) and the number of branching per larva (**f**). Data are expressed as mean ± SEM from 6 independent experiments, each with 15–20 larvae per experimental condition (**b**,**e**) and mean ± SEM from 90 to 100 larvae for each experimental condition, obtained from 6 independent experiments (**c**,**f**). Statistical analysis was performed by Student’s t-test. *p < 0.05 and ***p < 0.001 vs control. (**a**,**d**) Lateral views, anterior to the left; scale bar: 100 µm. Images were created using GraphPad PRISM version 6.0 (https://www.graphpad.com/support/), Photoshop version CS5 (https://www.adobe.com/products/photoshop.html) and PowerPoint version 16.16.24 (https://support.microsoft.com/en-us/powerpoint).

Previous results indicated that vascular alterations may be associated with pericardial edema in developing zebrafish^39^. To study the effects induced by BRB on vascularization, we examined the formation of two vascular structures typically analyzed in angiogenic studies, such as the sub-intestinal veins vessels (SIV, Fig. 6a) and the intersegmental vessels (ISVs, Fig. 6d), in 72 hpf zebrafish larvae previously exposed for 48 h to either control conditions or 100 mg/L BRB. The SIV is a basket of mostly venous vessels that starts to develop by ~ 48 hpf, extends on the surface of the yolk ball at ~ 72 hpf and progressively regresses by ~ 96 hpf^42^. By Alkaline Phosphatase staining we observed that 100 mg/L BRB induced an abnormal growth of newly formed vascular structures in the SIV basket (sprouting, black arrowheads, Fig. 6a). In particular, compared to control conditions, quantitative analysis showed that BRB induced a significant increase (~ 4.2-fold vs control) in the percentage of larvae with abnormal SIV development (Fig. 6b), as well as an increased number of SIV sprouting per larvae (~ 5.8-fold vs control) (Fig. 6c).

The ISVs are dorso-lateral vessels that originate early from the dorsal aorta and the posterior cardinal vein, interconnect dorsally and, by ~ 72 hpf, nearly all of them are functioning^29^. To investigate the effects of BRB on ISVs development, we examined the trunk region of transgenic Tg(*kdrl*:EGFP)^S843^/*roy*^*a9/a9*^*;nacre*^*w2/*w2^ casper larvae, observing that 100 mg/L BRB treatment promoted the development of anomalous ramifications, such as branching or protrusions in this region (red arrows, Fig. 6d). In particular, our quantitative analysis showed that compared to controls, the treatment with BRB induced a significant increase (~ 13.3-fold vs control) in the percentage of larvae with altered phenotype of ISVs (Fig. 6e) as well as an increased number of branching per larvae (~ 41.7-fold vs control) (Fig. 6f).

**Figure 7:**
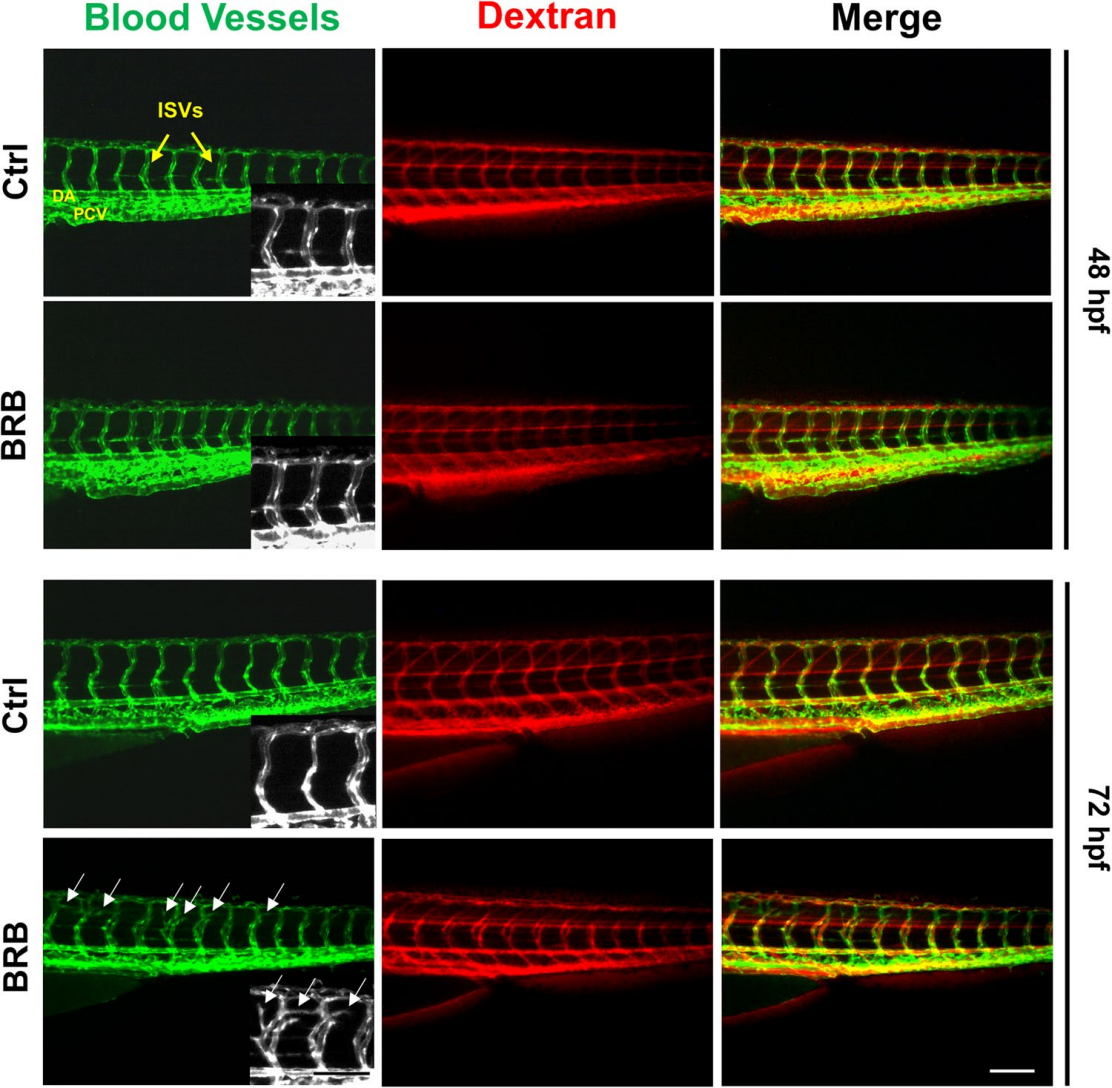
Effects of BRB on vascular permeability in developing zebrafish larvae. Representative images of microangiographies performed injecting Dextran-Texas Red 20 kDa in the venous sinus of Tg(*kdrl*:EGFP)^S843^/*roy*^*a9/a9*^*;nacre*^*w2/w2*^ casper zebrafish larvae at 48 and 72 hpf, previously incubated either in control conditions (Hank’s buffer; Ctrl) or in the presence of 100 mg/L BRB, for 24 and 48 h, respectively. Images show the absence of extravasation. White arrows indicate the abnormal branching of the ISV. *DA* dorsal aorta, *PCV* posterior cardinal vein. Lateral views, anterior to the left; scale bar: 100 µm. Images were created using Photoshop version CS5 (https://www.adobe.com/products/photoshop.html) and PowerPoint version 16.16.24 (https://support.microsoft.com/en-us/powerpoint).

To further investigate the vascular abnormalities promoted by BRB treatment, we examined the integrity of blood vessels using microangiography analysis. In this respect, zebrafish larvae at 48 and 72 hpf previously treated for 24 and 48 h respectively, either in control conditions or with 100 mg/L BRB, were injected with Dextran-Texas Red 20 kDa in venous sinus. As shown in Fig. 7, in line with control embryos, in BRB-treated embryos at both 48 and 72 hpf we did not observe extravasation of the fluorescent dye, suggesting that BRB exposure does not affect vascular permeability.

### Effects of BRB on *vegfaa*, *vegfab*, *cdh5* and prolyl hydroxylase 3 (*phd3*) mRNA expression in zebrafish larvae

**Figure 8:**
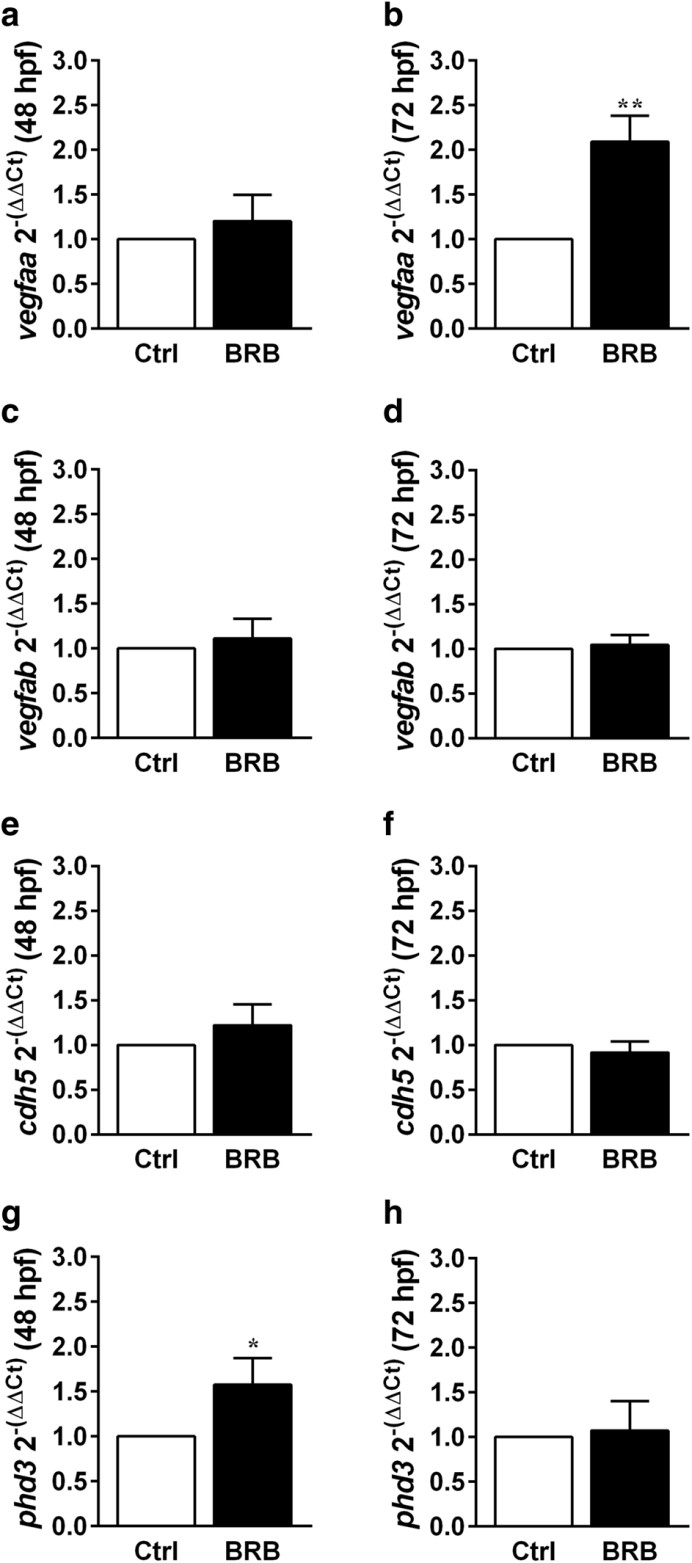
Effects of BRB on *vegfaa*, *vegfab*, *cdh5* and *phd3* mRNA expression in developing zebrafish. Evaluation of *vegfaa*, *vegfab*,*cdh5* and *phd3* mRNA levels by qRT-PCR in zebrafish at 48 hpf (**a**,**c**,**e**,**g**, respectively) and 72 hpf (**b**,**d**,**f**,**h**, respectively), previously exposed from 24 hpf to eihter control conditions (Hank’s buffer; Ctrl) or to 100 mg/L BRB. (**a**,**c**,**e**,**g**,**h**) Data are expressed as mean ± SEM from 4 independent experiments, each with 30 larvae per experimental condition. Statistical analysis was performed by Kruskal–Wallis test followed by Dunn’s multiple comparison test. (**b**,**d**,**f**) Data are expressed as mean ± SEM from 5 independent experiments, each with 30 larvae for each experimental condition. Statistical analysis was performed by one-way ANOVA, followed by Tukey post hoc test. *p < 0.05 and **p < 0.01 vs control. Images were created using GraphPad PRISM version 6.0 (https://www.graphpad.com/support/).

In larvae at 48 and 72 hpf, after the incubation either in control conditions or with 100 mg/L BRB for 24 and 48 h, respectively, we analyzed the expression level of *vegfaa*, *vegfab*, two zebrafish VEGF-A orthologs^39,43^, *cdh5*^32^ and *phd3*, a member of prolyl hydroxylase containing proteins involved in oxygen sensing, whose expression is induced by hypoxia in vertebrates^44^. Treatment with 100 mg/L BRB induced a significant increase of *vegfaa* expression in 72 hpf larvae (Fig. 8b) when compared to control conditions (~ 109% vs control), whereas no significant differences were observed at 48 hpf (Fig. 8a). In addition, BRB treatment did not significantly affect mRNA level of *vegfab* (Fig. 8c,d) and *cdh5* (Fig. 8e,f) at both developmental stages. Finally, as shown in Fig. 8g, *phd3* expression level was increased in BRB-treated 48 hpf embryos (~ 41% vs control) whereas resulted unaffected by BRB treatment at 72 hpf (Fig. 8h).

## Discussion

In the initial part of this work we confirm, and extend to the zebrafish *roy*^*a9/a9*^*;nacre*^*w2/*w2^ casper line, previous observations by Ali et al.^22–24^ reporting that BRB treatment promotes toxic effects and induces malformations, both in a time- and concentration-dependent manner, in wt zebrafish embryos of AB strain. In addition, we obtained LC_50_ values that are very close to those previously reported^22,23^, suggesting that the response to the toxic insult of BRB treatment observed in the *roy*^*a9/a9*^*;nacre*^*w2/w2*^ casper line is very similar to those described for the wt AB strain, and emphasize the consistency of the experimental protocol used. In the present study, we focused on cardiovascular effects of BRB treatment providing a detailed time-course of pericardial edema formation, showing that this morphological abnormality is observable from ~ 72 hpf, in agreement with the timing of the effects of BRB treatment on embryonic mortality. In this respect, it has been proposed that some developmental processes that take place between 48 and 72 hpf, such as changes in permeability of the gills or skin, and the spontaneous exit from the chorion, could make the larva more sensitive to chemical insults and/or facilitate the contact with the compound^23^. However, we cannot exclude that toxic and teratogenic effects of BRB on developing zebrafish embryos may stem, at least in part, from the obstruction of chorion pores, with a resulting blockade of oxygen transport from the medium into the embryo, as previously demonstrated, for instance, for graphene oxide and iron oxide nanoparticles^45,46^. The results of our wash-out experiments, showing that the percentage of embryos developing the pericardial edema continues to increase after BRB removal, suggest that BRB treatment may trigger an abnormal developmental program which irreversibly proceeds even in its absence. Additionally, BRB might accumulate in various organs and tissues, as well as in specific cell compartments^15,47,48^ and from these locations could maintain its influence on development progression.

In keeping with zebrafish results, a small number of studies in rodents, in vivo and in vitro, using various modalities of administration, showed that BRB may exert toxic and teratogenic effects during development^18–21^. On the other hand, discrepancies between our results and those indicating the lack of toxic and/or teratogenic effects of BRB during development^25–27^ could be due, at least in part, to the different experimental model, concentration/dose and route of administration of the compound, confirming previous observations^6^.

As an abnormal functionality and morphogenesis of the cardiovascular system is known to contribute to pericardial edema formation in fishes and other vertebrates^32,39–41,43,49^, we also investigated whether treatment with sub-lethal concentrations of BRB may affect cardiovascular functionality and morphogenesis in zebrafish embryos. In this respect, most of the cardiac electrical and contractile properties as well as the underlying molecular mechanisms in zebrafish are similar to those in other vertebrates, including humans^28,38^. To our knowledge, there are no available data on BRB effects on cardiac functionality during embryonic development. However, several data in vivo and in vitro*,* as well as clinical studies, demonstrated that BRB modulates the cardiovascular functionality with great effectiveness, acting through multiple mechanisms that impact on the electrical and contractile properties of the heart^5^. Generally, BRB is considered beneficial at cardiovascular level^5,17,50^ although data obtained in some experimental models^51^ and in a hypervagotonic patient^52^ indicate that BRB actions on cardiac functionality remain in part controversial. Our results, showing bradycardic effects and the reduction of cardiac output, atrial stroke volume and contractility in BRB-treated zebrafish embryos indicate, for the first time, that BRB may severely impair the cardiac functionality from the earlier stages of embryonic development. In keeping with our results, Hu and colleagues showed that the BRB-type natural alkaloid Coptisine counteracts the increased heart rate promoted by 2, 2′-azobis [2-methylpropionamidine] dihydrochlorid exposure in developing zebrafish embryos^53^. The impairment of cardiac function we observed in BRB-treated animals may reveal a peculiar sensitivity of the embryonic stages to the cardiotoxic insults of BRB. Additionally, we explored whether the bradycardic effect of BRB treatment on zebrafish embryos may be associated with a type of atrioventricular blockade due to prolonged QT length, which may be experimentally revealed in zebrafish by A/V rates ratio values other than 1:1^37^. However, our results show that A/V rates ratio remains 1:1 in BRB-treated animals suggesting that sub-lethal concentrations of BRB do not promote atrioventricular uncoupling. As a reduced cardiac output and contractility are the first defects that trigger pericardial edema formation in zebrafish embryos treated with various unrelated chemical compounds^40^, we propose that the impairment of the cardiac functionality may be a pivotal event also in pericardial edema formation elicited by BRB treatment.

The morphogenesis of the heart in zebrafish, which includes various processes, such as the cardiac looping, shares many features in common with other vertebrates^28,38^. Our results, which to our knowledge have not been previously reported, show an increased incidence of embryos displaying a linear heart tube after BRB treatment at 48 and 120 hpf, suggesting that BRB treatment may impair cardiac looping during early phases of cardiac morphogenesis. Furthermore, there is evidence that cardiac looping defects may be triggered by a wide variety of insults and experimental manipulations, and may be involved in pericardial edema formation in vertebrates^32,39–41,54^. Therefore, our results suggest that the impaired cardiac looping may play a role in pericardial edema formation promoted by BRB treatment. On the contrary, our data indicate that the formation of cardiac valves, whose improper development could play a role in the appearance of pericardial edema in vertebrates^39,49^, does not seem to be affected by BRB exposure.

Previous data showed that alterations in the reciprocal chemical-physical interaction between myocardium and endocardium may affect their morphological and functional properties in vertebrates^28,31^, sometimes leading to an abnormal myocardial-endocardial separation^32,49^. Therefore, the increased incidence of a persistent myocardial-endocardial detachment in the atrium of BRB-treated zebrafish embryos we observed at 120 hpf, may indicate that the alkaloid interferes with the crosstalk between myocardial and endocardial cells. Interestingly, the atrial myocardial-endocardial separation observed at later stages in developing zebrafish embryos appears to coherently correlate with the reduced atrial contractility we reported during the earlier stages of development. As BRB was shown to influence myocardial cells, acting through the regulation of several molecular pathways^10,11^, it is possible to speculate that effects on this cell type may result, in turn, in the myocardial-endocardial detachment we observed. Finally, since the separation between these tissues may play an important role in various cardiac alterations in vertebrates, including the formation of pericardial edema^32,49^, it is possible to hypothesize that myocardial-endocardial detachment may play a role in pericardial edema development that we observed in BRB-treated embryos.

Zebrafish vascular development shares high similarity with other vertebrates and represents an excellent tool to study the vascularization process in physiological and pathological conditions^29^. A large amount of data in vivo and in vitro indicated that BRB may exert anti-angiogenic action; however, information on BRB effects on embryonic vascularization are very limited^14,35,36^. As we find that BRB treatment from 72 hpf causes an increased and abnormal angiogenesis in both ISVs and SIV during embryonic development, we hypothesize that discrepancies between our results and those previously reported may be due, at least in part, to the different experimental protocol used, BRB concentration, formulation and administration time. In this respect, some compounds may exert pro- or anti-angiogenic effects depending on their concentration^55^ or on the developmental stage of treated embryos/fetuses^24,27^. Additionally, since an aberrant vascularization may be involved in the genesis of pericardial edema in vertebrates^39,49^, we hypothesize that the altered angiogenesis we observed may also play a role in the pericardial edema formation in BRB-treated developing embryos.

Finally, our results showing no extravasation in BRB-treated animals indicate that this natural compound may affect the angiogenic process without affecting vascular permeability during embryonic development, in line with results previously reported in VEGF-treated quail embryos^56^. In addition, results in vivo in rodents and in vitro in rat microvascular endothelial cells indicated that BRB may counteract vascular permeability^33,57,58^.

VEGF-A plays a critical role in the development and functionality of the cardiovascular system in vertebrates, both in physiological and pathological conditions^29–31^. In zebrafish, two homologues of mammalian VEGF-A have been described: *vegfaa* and *vegfab*, encoding for efficiently secreted and poorly secreted isoforms, respectively^39,43^. Herein, BRB induces an increased level of *vegfaa* mRNA at 72 hpf, while *vegfab* mRNA level was unaffected, suggesting a possible primary role of *vegfaa* as a mediator of BRB vascular effects and in agreement with previous observations indicating that *vegfaa* and *vegfab* may play slightly different roles during vascular development^43^. It is worth noting that BRB-induced *vegfaa* expression appears as an unexpected result since a large amount of data in vitro and in vivo in mice previously demonstrated that BRB exerts inhibitory effects on VEGF-A and its signaling^12–14^. To explain this apparent contradiction, it is possible to hypothesize that the increased *vegfaa* expression may be an indirect effect of BRB treatment resulting from an inadequate blood flow, due to defective cardiovascular functionality and/or morphogenesis, causing changes of shear stress, decreased transport of nutrients and oxygen to tissues that, in turn, promote the VEGF-A increase^59,60^. Indeed, VEGF-A is a target gene of the hypoxia inducible factor-1 alpha (HIF1-α), one of the main transcription factors that regulate the response to hypoxia, and notoriously induced by oxygen depletion^61^. In the same line, we also reported an increased mRNA level of *phd3*, whose expression is positively regulated by HIF1-α in vertebrates, and is a well-known marker associated with hypoxia^44^. Curiously, the lack of effects on vascular permeability reported in BRB-treated embryos seems to disagree with the increased *vegfaa* levels, as VEGF-A is also known to promote vascular permeability^30^. However, previous data in mouse brain suggest the possible existence of a threshold below which the increase in VEGF-A mRNA and protein may not produce changes in vascular permeability^62^.

On the other hand, *vegfaa* upregulation observed at 72 hpf may have a role in myocardial-endocardial detachment we described at 120 hpf. Indeed, VEGF-A is one of the most tightly controlled key signal produced by myocardial cells, critically involved in the reciprocal crosstalk between myocardium and endocardium and a dysregulation in its expression seems to be sufficient to alter this process^31,49,56,60^.

Our results also show that BRB treatment does not affect the mRNA level of *cdh5*, a gene whose knockdown causes impaired cardiac looping and myocardial-endocardial detachment^32^. These data suggest that Cdh5 regulation may not be a critical target of the developmental defects promoted by BRB treatment in zebrafish. On the other hand, we emphasize that our hypothesis on the possible involvement of Vegfa, but not Cdh5, as mediator of BRB treatment, is strictly related to RNA expression analysis performed on whole embryos, therefore not being able to detect potential district-specific differences, as well as post-transcriptional and/or post-translational modulations that may occur.

In conclusion, we showed that BRB may exert developmental toxicity and affect various aspects of the cardiovascular system morphogenesis and functionality, which in zebrafish may all contribute to the formation of an abnormal pericardial edema. In addition, we provide novel information further supporting the idea that BRB treatment during pregnancy and lactation should be avoided or at least considered with extreme caution and underlie the importance of a continuous research on BRB effects during embryonic development.

## Methods

### Zebrafish care

Animal procedures were performed in strict compliance with protocols approved by Italian Ministry of Public Health and the local Ethical Committee of the University of Pisa (authorization n. 99/2012-A, 19.04.2012), in conformity with the Directive 2010/63/EU. Zebrafish casper (*roy*^*a9/a9*^*;nacre*^w2/w2^) and Tg(*kdrl*:EGFP)/casper (transgenic line generated by crossing Tg(*kdrl*:EGFP)^S843^ fishes with casper fishes) embryos were obtained by natural mating and maintained at 28 °C in E3 zebrafish medium 1X (NaCl 5 mM, KCl 0.17 mM, CaCl_2_ 0.33 mM, MgSO_4_ 0.35 mM; pH 7.2).

### BRB treatment

In line with previous works^22–24^, BRB chloride (C_20_H_18_ClNO_4_, molecular weight 371.81; B3251, Sigma Aldrich, St. Louis, MO, USA) solutions were freshly prepared in Hank’s balanced salt solution (Hank’s buffer without sodium bicarbonate; H6136-10X, Sigma Aldrich) at a concentration of 0.98 g/L in Milli-Q water, with the addition of sodium bicarbonate at 0.035 g/L (S5761, Sigma Aldrich), and adjusted to pH 7.4. 24 hpf zebrafish embryos were transferred into 96-well microtitre plates (Sarstedt, Nuembrecht, Germany), dispensing 1 embryo/well in 250 μL of solution and maintained in the dark at 28 °C for the indicated period of exposure. Control embryos were maintained in buffered Hank’s solution.

### Evaluation of toxicological and teratogenic effects of BRB on zebrafish development

The toxicity tests were performed in line with previous studies^22–24^, chronically exposing zebrafish embryos to control conditions or increasing concentrations of BRB (50, 100, 200 and 400 mg/L), applied for different exposure durations and starting from various developmental stages between 24 (to avoid early embryos mortality) and 120 hpf^22^.

The survival rate and teratogenic response, with particular focus on pericardial edema formation, of embryos/larvae were monitored and observed at different developmental stages using a dissecting stereomicroscope. Consistently with previous works^22–24^, embryos/larvae were considered dead if they were no longer moving and the heart was not beating. Where reported, the LC_50_ dose expressed in mg/L was determined based on cumulative mortality using Regression Probit analysis.

### Whole mount in situ hybridization

WISH was performed essentially as previously described^63^. To generate antisense RNA probes, templates for *cmlc2* (*myl7)*, *bmp4* and *notch1b* genes were transcribed in vitro using either SP6 or T7 RNA polymerase and DIG RNA labeling Mix (Roche Diagnostics GmbH, Mannheim, Germany) following manufacturer’s instructions.

Zebrafish embryos at 48 hpf and 72 hpf were exposed to control conditions or 100 mg/L BRB for 24 and 48 h, respectively, and successively fixed with 4% paraformaldehyde (PFA) in PBS for 1 h at room temperature (RT), dehydrated gradually with methanol/PBS series (25, 50 and 75%) and stored at − 20 °C. Images were acquired using a stereomicroscope Nikon SMZ1500.

### Histological studies

Embryos at 120 hpf, previously exposed to control conditions or 100 mg/L BRB for 96 h, were fixed in 4% PFA in PBS, dehydrated through an ethanol series (100, 95, 70%), cleared in xylene and embedded in paraffin wax. 5 μm thick longitudinal sections were cut, dewaxed in xylene, dried and stained with Hematoxylin (Sigma Aldrich) and Eosin (Merck Millipore, Darmstadt, Germany) solution (H&E). Histological sections were photographed at cardiac level using Nikon eclipse E600.

### Cardiac function analysis

Once anesthetized (0.16 mg/mL of tricaine), 48 hpf zebrafish embryos of the Tg(*kdrl*:EGFP)^S843^/*roy*^*a9/a9*^*;nacre*^*w2/w2*^ casper line, previously exposed for 24 h in control conditions or 100 mg/L BRB, were singularly transferred in a 35 mm Petri dish and immobilized by 1% low melting agarose in the presence of E3 medium 1X containing tricaine. Experiments were performed as described elsewhere^64^. As muscle contraction resulted in a higher fluorescence emission, the heart rate (beats/sec) was measured in the graph displaying the periodical changes of signal in selected regions of interest. From the analysis of the 7.92 s video we evaluated the atrial and ventricular beat rates that were used to calculate the atrial-to-ventricular (A/V) rate ratio. Additionally, from the same video, and in line with the method indicated in a previous work^65^ we also calculated the stroke volume (volume of blood pumped per beat; nL), cardiac output (volume of blood pumped from the ventricle in a min given by the product of the heart rate and the ventricular stroke volume; nL/min) and shortening fraction % (index of muscular contractility, which is expressed as percentage of the difference between end-diastolic diameter and end-systolic diameter over end-diastolic diameter). The data were obtained using Imaging Workbench 2.1 (https://www.imagingworkbench.com/), and Image J software (https://imagej.nih.gov/ij/download.html) or routines developed for the scope in LabVIEW 8.2.

### Whole-mount alkaline phosphatase vessel staining

Zebrafish larvae at 72 hpf, previously exposed to control conditions or 100 mg/L BRB for 48 h, were fixed in 4% PFA in PBS for 2 h at RT and stained for endogenous alkaline phosphatase, in line with a previous work^66^. Briefly, after fixation, larvae were washed 3 times in PBS containing 0.1% Tween-20 (PBST). Then, larvae were dehydrated and made permeable with ethanol/PBST series (30, 50 and 70%) for 10 min/each and finally stored in 100% ethanol at − 20 °C. Larvae were then rehydrated with ethanol/PBST series (70, 50 and 30%) for 10 min/each and finally resuspended in PBST. Embryos were equilibrated with alkaline phosphatase buffer NTMT (0.1 M Tris–HCl pH 9.5; 50 mM MgCl_2_; 0.1 M NaCl; 0.1% Tween 20) for 15 min at RT and then incubated in staining solution (NBT/BCIP, Roche Diagnostics) for 10 min. The staining reaction was stopped washing larvae with PBST for 3 times. Stained larvae were fixed in 4% PFA in PBS for 2 h at RT and then stored in 100% ethanol at − 20 °C. Successively, larvae were photographed using a stereomicroscope Nikon SMZ1500.

### Microangiography

Zebrafish larvae of the Tg(*kdrl*:EGFP)^S843^/*roy*^*a9/a9*^*;nacre*^*w2/w2*^ casper line at 48 and 72 hpf, previously exposed to control conditions or 100 mg/L BRB for 24 and 48 h, respectively, were anesthetized with 0.16 mg/mL of tricaine (Sigma Aldrich), placed in agarose and injected into the venous sinus with 1 nL of Dextran-Texas Red 10,000 MW (D1828, Invitrogen) in PBS (8 mg/mL final concentration). After injection of Dextran-Texas Red, larvae were transferred to E3 1X zebrafish medium and after 15 min anesthetized and then imaged by a stereomicroscope Nikon SMZ1500.

### RNA extraction and Real-time quantitative RT-PCR assay

Total RNA was extracted from 30 embryos at 48 and 72 hpf, previously exposed to control conditions or 100 mg/L BRB, for 24 and 48 h respectively, using Trizol reagent (Invitrogen, Carlsbad, CA, USA) and RNeasyMinikit (Qiagen, Venlo, The Netherlands). Extracted RNA was quantified using NanoDrop-1000 spectrophotometer and the cDNA was obtained using QuantiTect Reverse Transcription kit (Qiagen), following the manufacturer's protocol. *vegfaa*, *vegfab cdh5, phd3* and *β-actin* (as internal control) expression levels were evaluated by quantitative reverse transcription-polymerase chain reaction (qRT-PCR) using the SYBR Green method (SensiMix SYBR kit, Bioline, London, UK), following the manufacturer's protocol. Real time PCR and relative quantification of each gene expression was performed essentially as previously described^67^. Primers used for qRT-PCR are listed in Supplementary Table S3. Transcript level of examined genes was normalized to β-actin mRNA level according to standard procedures.

### Statistical analysis

After verification of the normal distribution of the data by Shapiro–Wilk and/or Kolomogorov Smirnov test, statistical analysis was performed with Student’s *t*-test, Fisher exact test or one-way analysis of variance (ANOVA) followed by the Tukey post hoc test. Mann–Whitney U test or Kruskal–Wallis test followed by Dunn’s multiple comparison test were applied when data were not normally distributed. The software GraphPad PRISM version 6.0 (https://www.graphpad.com/support/ GraphPad, San Diego, CA, USA) was used for data analysis and image creation; a value of *p* < 0.05 was considered significant.

## Supplementary information

Supplementary Information.

## Data Availability

The datasets generated during and/or analysed during the current study are available from the corresponding author on reasonable request.
