# Supplementary material for: Exposure to the natural alkaloid Berberine affects cardiovascular system morphogenesis and functionality during zebrafish development
Source: Sci Rep. 2020 Oct 15;10:17358. doi: 10.1038/s41598-020-73661-5 (PMC7566475; doi:10.1038/s41598-020-73661-5)
Supplement: Supplementary file 1 — Supplementary Information. [file 41598_2020_73661_MOESM1_ESM.pdf]

TITLE:

Exposure to the natural alkaloid Berberine affects cardiovascular system morphogenesis and functionality during zebrafish development

DAVIDE MARTINI[1#], CECILIA PUCCI[1,2 #,§], CHIARA GABELLINI[1], MARIO PELLEGRINO [3] and MASSIMILIANO ANDREAZZOLI[*,1,4]

[1]*Cell and Developmental Biology Unit, Department of Biology, University of Pisa, Pisa, Italy*

[2]*Sant'Anna School of Advanced Studies, Pisa, Italy*

[3]*National Institute of Optics, National Research Council, Pisa, Italy*

[4]*Interdepartmental Research Center Nutrafood "Nutraceuticals and Food for Health", University of Pisa, Pisa, Italy*

[#] These authors equally contributed to this work.

[§]Present address: Institute of Genomic Medicine, Catholic University, 00168 Rome, Italy.

[*] Corresponding author:

massimiliano.andreazzoli@unipi.it

Cell and Developmental Biology Unit, Department of Biology, University of Pisa SS12 Abetone e Brennero, 56127 Pisa, Italy

Tel. +39-050-2211485; FAX + 39-050-2211495

Supplementary Table S1

Percentage of mortality at different timepoints and BRB concentrations

	24 h	48 h	72 h	96 h
**control**	0.5 ± 0.5	1.3 ± 0.83	2.28 ± 0.64	2.75 ± 0.81
**50 mg/L**	0.98 ± 0.6	2.25 ± 0.99	2.75 ± 0.81	2.75 ± 0.81
**100 mg/L**	0.98 ± 0.6	3.08 ± 1.32	8.33 ± 1.21	(***)(###)<br>25.16 ± 5.06
**200 mg/L**	3.08 ± 1.54	5.65 ± 2.74	(***)(###)($$$)<br>42.13 ± 10.12	(***)(###)($$$)<br>87.24 ± 4.95
**400 mg/L**	4.88 ± 1.85	(***)(###)($$$)<br>36.74 ± 3.67	(***)(###)($$$)(ϕϕϕ)<br>95.27 ± 2.36	(***)(###)($$$)<br>100 ± 0.0

Data are cumulative and expressed as mean ± standard error of the mean (SEM) from 5 independent experiments, each with 40 larvae per experimental condition. One-way ANOVA followed by Tukey post hoc test. Statistical significance refers to the same exposure time, in the various experimental conditions. ***p<0.001 vs control, $^{\#\#\#}p$<0.001 vs 50 mg/L, $^{\$\$\$}p$<0.001 vs 100 mg/L, $^{\phi\phi\phi}p$<0.001 vs 200 mg/L.

Supplementary Table S2

Percentage of embryos with pericardial edema at different timepoints and BRB concentrations

	**24 h**	**48 h**	**72 h**	**96 h**
**control**	0.00	0.192 ±0.192	0.5448 ± 0.29	0.5448 ± 0.29
**50 mg/L**	0.00	1.34 ± 0.73	3.947 ± 0.90	6.38 ± 1.57
**100 mg/L**	0.00	(*)(#)<br>12.14 ± 2.64	(***)(###)<br>26.67 ± 2.93	(***)(###)<br>54.88 ± 5.01
**200 mg/L**	0.00	(***)(###)<br>25.17 ± 8.65	(***)(###)($)<br>40.81 ± 6.74	(***)(###)<br>40.81 ± 6.74
**400 mg/L**	1.82±1.82	(***)(###)($)<br>27.69 ± 7.27	(***)(###)<br>27.69 ± 7.27	(***)(#)($$$)<br>27.69 ± 7.27

Data are cumulative and expressed as mean ± SEM from 5 independent experiments, each with 40 larvae per experimental condition. One-way ANOVA followed by Tukey post hoc test. Statistical significance refers to the same exposure time, in the various experimental conditions:
$^{*}p<0.05$ vs control, $^{***}p<0.001$ vs control, $^{\#}p<0.05$ vs 50 mg/L, $^{\#\#\#}p<0.001$ vs 50 mg/L,
$^{\$}p<0.05$ vs 100 mg/L, $^{\$\$\$}p<0.001$ vs 100 mg/L.

Supplementary Table S3

Sequences of primers used in qRT-PCR

**Gene name**		**Sequences**
cdh5	**F**	5' - TGGAATGAGTGTCAGTGCCC - 3'
	**R**	5' - CAGCTGCTCATGGATTTCGC - 3'
vegfaa	**F**	5' - AAAAGAGTGCGTGCAAGACC - 3'
	**R**	5' - GACGTTTCGTGTCTCTGTCG - 3'
vegfab	**F**	5' - TGTTGGTGGAAATTCAGCAG - 3'
	**R**	5' - CACCCTGATGACGAAGAGGT - 3'
phd3	**F**	5’ - CCTGGAAATGGAGCTGGATA - 3’
	**R**	5’ – CCGGTCAAATAAAGGCTCAA - 3’
β-actin	**F**	5' - CGAGCAGGAGATGGGAACC - 3'
	**R**	5' - CAACGGAAACGCTCATTGC - 3'
